# Glucocorticoid Receptor Maintains Vasopressin Responses in Kidney Collecting Duct Cells

**DOI:** 10.3389/fphys.2022.816959

**Published:** 2022-05-24

**Authors:** Hsiu-Hui Yang, Shih-Han Su, Cheng-Hsuan Ho, Ai-Hsin Yeh, Yi-Jiun Lin, Ming-Jiun Yu

**Affiliations:** Institute of Biochemistry and Molecular Biology, College of Medicine, National Taiwan University, Taipei, Taiwan

**Keywords:** vasopressin, aquaporin-2, glucocorticoid receptor, TGFβ, TNF, collecting duct

## Abstract

Water permeability of the kidney collecting ducts is regulated in part by the amount of the molecular water channel protein aquaporin-2 (AQP2), whose expression, in turn, is regulated by the pituitary peptide hormone vasopressin. We previously showed that stable glucocorticoid receptor knockdown diminished the vasopressin-induced *Aqp2* gene expression in the collecting duct cell model mpkCCD. Here, we investigated the pathways regulated by the glucocorticoid receptor by comparing transcriptomes of the mpkCCD cells with or without stable glucocorticoid receptor knockdown. Glucocorticoid receptor knockdown downregulated 5,394 transcripts associated with 55 KEGG pathways including “vasopressin-regulated water reabsorption,” indicative of positive regulatory roles of these pathways in the vasopressin-induced *Aqp2* gene expression. Quantitative RT-PCR confirmed the downregulation of the vasopressin V2 receptor transcript upon glucocorticoid receptor knockdown. Glucocorticoid receptor knockdown upregulated 3,785 transcripts associated with 42 KEGG pathways including the “TNF signaling pathway” and “TGFβ signaling pathway,” suggesting the negative regulatory roles of these pathways in the vasopressin-induced *Aqp2* gene expression. Quantitative RT-PCR confirmed the upregulation of TNF and TGFβ receptor transcripts upon glucocorticoid receptor knockdown. TNF or TGFβ inhibitor alone, in the absence of vasopressin, did not induce *Aqp2* gene transcription. However, TNF or TGFβ blunted the vasopressin-induced *Aqp2* gene expression. In particular, TGFβ reduced vasopressin-induced increases in Akt phosphorylation without inducing epithelial-to-mesenchymal transition or interfering with vasopressin-induced apical AQP2 trafficking. In summary, our RNA-seq transcriptomic comparison revealed positive and negative regulatory pathways maintained by the glucocorticoid receptor for the vasopressin-induced *Aqp2* gene expression.

## Introduction

Aquaporin-2 (AQP2) is a molecular water channel protein expressed in the kidney collecting duct principal cells responsible for osmotic water reabsorption (Knepper et al., 2015). AQP2 is regulated by the pituitary peptide hormone vasopressin chiefly in two modes. In the short-term response (minutes to hours), vasopressin induces dynamic changes in the cortical actin filaments to facilitate the fusion of AQP2-containing vesicles with the apical plasma membrane of the cells (Simon et al., 1993; Loo et al., 2013). This increases the amount of apical AQP2 and hence water permeability (Nielsen et al., 1995; Yamamoto et al., 1995). In the long-term response (hours to days), vasopressin increases the *Aqp2* gene expression (DiGiovanni et al., 1994). Vasopressin regulates both responses by signaling through vasopressin V2 receptor, Gαs, adenylyl cyclase 6, and cAMP that activates two PKA catalytic subunits (PKA-Cα and PKA-Cβ) (Judith Radin et al., 2012; Pearce et al., 2015; Isobe et al., 2020). Dysregulations in either response cause a number of water balance disorders (Noda et al., 2010; Judith Radin et al., 2012; Moeller et al., 2013). Understanding both responses is critical to the physiology and pathophysiology of the disorders. Both responses have been actively studied, although less is known about the latter response, that is, vasopressin-regulated *Aqp2* gene transcription.

Gene expression often involves the binding of transcription factors to the promoter or enhancer regions upstream or downstream to the target gene (Yu et al., 2009). Through promoter-reporter assays, several transcription factors have been implicated in *Aqp2* gene transcription such as Creb1 (Hozawa et al., 1996; Yasui et al., 1997), Elf3 (Yu et al., 2009; Lin et al., 2019), Elf5 (Yu et al., 2009; Grassmeyer et al., 2017), Ehf (Yu et al., 2009), Gata-3 (Uchida et al., 1997), and Nfat5 (Hasler et al., 2006). These transcription factors were studied in various cell models that often do not express endogenous vasopressin V2 receptor or AQP2, making it challenging to compose a comprehensive *Aqp2* gene transcription network. For example, Creb1 has been the primary transcription factor for *Aqp2* gene transcription in many review studies (Nielsen et al., 2002; Bockenhauer and Bichet, 2015; Pearce et al., 2015). Recent ChIP-seq analysis showed the indirect involvement of Creb1 in *Aqp2* gene transcription (Jung et al., 2018). A number of other transcription factor candidates were suggested for future investigation (Kikuchi et al., 2021), preferentially with CRISPR/Cas9-based gene knockout (Isobe et al., 2017; Datta et al., 2020; Isobe et al., 2020) or small hairpin RNA-mediated gene knockdown when gene knockout is lethal (Wang et al., 2017; Kuo et al., 2018; Lin et al., 2019; Wang et al., 2020; Wong et al., 2020).

Glucocorticoid receptor agonist betamethasone has been shown to enhance the vasopressin-induced *Aqp2* gene expression in infant rats within 6 h of injection (Yasui et al., 1996). To avoid influence from endogenous adrenal corticosteroids (i.e., glucocorticoid and mineralocorticoid), adrenalectomy is often performed before the administration of mineralocorticoid with or without dexamethasone (another glucocorticoid receptor agonist). In one study, the AQP2 protein levels were higher in the rats administered with dexamethasone and mineralocorticoid than those administered with the mineralocorticoid alone (Chen et al., 2005). In another study with similar settings, the AQP2 mRNA and protein levels were both lower in the rats administered with dexamethasone and mineralocorticoid than those administered with the mineralocorticoid alone (Saito et al., 2000). The latter observation could be explained *via* a suppressive effect of dexamethasone on the vasopressin gene promoter activity in the hypothalamic cells (Kim JK et al., 2001). The dexamethasone-suppressed vasopressin gene expression in the hypothalamus would, in turn, reduce the *Aqp2* gene expression in the collecting ducts.

We recently found that dexamethasone enhanced the vasopressin-induced *Aqp2* gene expression in a time- and dose-dependent manner in the mpkCCD cells, a collecting duct principal cell model that expresses all necessary molecular components for vasopressin signaling, *Aqp2* gene expression, and trafficking (Yu et al., 2009; Rinschen et al., 2010; Xie et al., 2010; Khositseth et al., 2011; Schenk et al., 2012; Loo et al., 2013; Kuo et al., 2018). Glucocorticoid receptor knockdown blunted the vasopressin-induced *Aqp2* gene expression in the cells (Ho et al., 2021). The effects of the glucocorticoid receptor involve α-actinin 4, an actin-bundling protein and a transcription co-activator of the glucocorticoid receptor (Honda, 2015; Zhao et al., 2017). α-Actinin-4 thus serves as a molecular link between vasopressin short-term responses in AQP2 trafficking and long-term responses in the *Aqp2* gene expression. It should be noted that the previous observations were made in the mpkCCD cells with stable glucocorticoid receptor knockdown, which could result in changes in the transcriptome landscape and thereby renders irresponsiveness to vasopressin. To test this, the present study used RNA-seq to compare transcriptomes of the stable glucocorticoid receptor knockdown vs. control cells. Genes that are downregulated in the glucocorticoid receptor knockdown cells are thought to have positive regulatory roles in the vasopressin-induced *Aqp2* gene expression. Genes that are upregulated in the glucocorticoid receptor knockdown cells are thought to have negative regulatory roles in the vasopressin-induced *Aqp2* gene expression.

## Materials and Methods

### Cell Culture

The mpkCCD cells re-cloned from their original line (Huyen et al., 1998) for the highest *Aqp2* gene expression level were maintained in the DMEM/Ham’s F-12 medium (DMEM/F-12, Cat. 11320033, Thermo-Fisher, United States) containing 2% fetal bovine serum (FBS) and supplements as described previously (Yu et al., 2009). The cells between 18 and 32 passages were grown on membrane supports (Transwell^®^, 0.4 μm pore size, Corning Costar, United States) prior to the experiments. FBS and supplements except for dexamethasone (Kuo et al., 2018) were removed from the medium to facilitate cell polarization (transepithelial electrical resistance >5,000 Ωxcm^2^ measured with an EVOM2 Epithelial Volt/Ohm Meter, World Precision Instruments, United States) before the cells were exposed to the vasopressin V2 receptor-specific agonist dDAVP (1-deamino-8-D-arginine vasopressin) in the basal medium to induce the endogenous *Aqp2* gene expression. In certain experiments, the cells were also exposed to TGFβ1 (Cat. 5231) or TNFα (Cat. 5178) from Cell Signaling, United States, or the TGFβ receptor inhibitor GW788388 (Cat. SML0116) or TNF receptor inhibitor SPD304 (Cat. S1967) from Sigma-Aldrich, United States. The HEK293T cells used for packaging small hairpin RNA (shRNA)-carrying lentivirus were maintained in the DMEM (Cat. 12491015, Thermo-Fisher, United States) containing 10% FBS.

### Small Hairpin RNA-Mediated Gene Knockdown

Small hairpin RNA (shRNA)-mediated glucocorticoid receptor (GR) knockdown was conducted *via* lentivirus-based transduction. The clones for shRNA were purchased from the National RNAi Core Facility, Academia Sinica, Taiwan: shCtrl (TRCN0000208001, control), shGR1 (TRCN0000238464), or shGR2 (TRCN0000238463). To produce shRNA-carrying lentivirus, the HEK293T cells were seeded at 70% confluence in a 60-mm dish. On the day of transfection, the medium was replaced with fresh DMEM containing 1% BSA excluding FBS and incubated for 30 min before transfection with a lentivirus-packaging plasmid mixture: 4 μg shRNA plasmid, 3.6 μg pCMVΔ8.91 plasmid, and 0.4 μg VSVG plasmid mixed in 250 μl Opti-MEM (Cat. 31985070, Thermo-Fisher, United States) and 12 μl T-Pro NTR II (Cat. JT97-N002M, T-Pro Biotechnology, Taiwan). Just two days after the transfection, the medium that contained lentiviral particles was collected and centrifuged at 1,200 x g for 5 min. The supernatants that contained shRNA-carrying lentiviral particles were aliquoted and stored at −80°C until use. To knockdown genes, 6 × 10^5^ mpkCCD cells were seeded in a 60-mm dish 1 day before infection with 1 ml lentivirus-containing medium with 2 ml regular medium and 24 μl polybrene (hexadimethrine bromide, Cat. H9268, Sigma-Aldrich, United States, 1 mg/ml) for 1 day. The infected cells were selected for stable knockdown with puromycin (Watson Biotechnology, Taiwan, 2.5 μg/ml) for two passages. GR mRNA was reduced to 43% (shGR1) and 45% (shGR2) of that in the non-target control, similar to previous values (Ho et al., 2021).

### RNA Extraction and Reverse Transcription

To each membrane support (12-mm Transwell^®^, Corning Costar, United States), 300 μl TRIzol^TM^ reagent (Cat. 15596081, Invitrogen, United States) was added to lyse the cells. Total RNA was then extracted using the RNA extraction kit (Cat. E1011-A, ZYMO Research, United States). About 500 ng total RNA was reverse transcribed to cDNA with the oligo (dT)_20_ primer (Cat. 18418020, Invitrogen, United States) or random hexamers (Cat. N8080127, Invitrogen, United States) using the SuperScript™ IV First-Strand Synthesis System (Cat. 18091050, Invitrogen, United States), following the manufacturer’s instruction.

### Quantitative Real-Time Polymerase Chain Reaction

Quantitative polymerase chain reaction (qPCR) was carried out using the SensiFAST SYBR^®^ Hi-ROX Kit (Cat. BIO-92005, Bioline, United States) with gene-specific intron-spanning primers (Table 1) in 8-strip qRT-PCR tubes. The qPCR program was performed in a thermal cycler (StepOnePlus™ Real-Time PCR Systems, Thermo-Fisher, United States) with the following steps: 1) polymerase activation (95°C, 3 min); 2) denaturation (95°C, 5 s); 3) annealing/extension (60°C, 30 s); 4) repeat (step 2–3, 40 cycles).

**TABLE 1:** Primers used in this study.

Gene symbol	Protein name	Primer sequence
*Acta2*	α-SMA	Forward: TGC​TGA​CAG​AGG​CAC​CAC​TGA​A
		Reverse: CAG​TTG​TAC​GTC​CAG​AGG​CAT​AG
*Adcy6*	Adenylyl cyclase 6	Forward: GCG​GTG​AGG​GAG​AAT​CAC​TG
		Reverse: TCA​CAC​CTG​TTA​CCT​CAC​GC
*Aqp2*	Aquaporin 2	Forward: CCT​CCT​TGG​GAT​CTA​TTT​CA
		Reverse: CAA​ACT​TGC​CAG​TGA​CAA​CT
*Avpr2*	V2 receptor	Forward: GACCGAGACCCGCTGTTA
		Reverse: CGA​CCC​CGT​CGT​ATT​AGG​G
*Creb3*	Creb3	Forward: TGC​GCG​GAG​GGA​TTT​CTA​TC
		Reverse: CCA​CCC​GAA​GGC​CTA​TCA​C
*E2f4*	E2F4	Forward: CCC​ATC​CCA​GAG​GGT​CTC​AA
		Reverse: TGT​TCA​CTA​GCA​GCA​CCT​CG
*Gnas*	Gαs	Forward: ATG​GGT​TTA​ACG​GAG​AGG​GC
		Reverse: GTC​CTG​CAC​TTT​AGT​GGC​CT
*Map3k7*	TAK1	Forward: CGT​GGC​GAC​TGC​AGG​TAA​C
		Reverse: TCT​GAC​ACT​AGG​GCT​GGA​TGA
*Mapk1*	ERK1	Forward: TCT​TAA​ATT​GGT​CAG​GAC​AAG​GG
		Reverse: AAG​AGT​GGG​TAA​GCT​GAG​ACG
*Nr3c1*	GR	Forward: GAC​TCC​AAA​GAA​TCC​TTA​GCT​CC
		Reverse: CTC​CAC​CCC​TCA​GGG​TTT​TAT
*Rela*	NFkB p65	Forward: CCT​CTG​GCG​AAT​GGC​TTT​AC
		Reverse: GAG​GGG​AAA​CAG​ATC​GTC​CA
*Rplp0*	60S Acidic ribosomal protein P0	Forward: AGA​TCG​GGT​ACC​CAA​CTG​TT
		Reverse: GGC​CTT​GAC​CTT​TTC​AGT​AA
*Smad3*	Smad3	Forward: AAG​AAG​CTC​AAG​AAG​ACG​GGG
		Reverse: CCA​TCC​AGT​GAC​CTG​GGG​AT
*Tgfbr1*	TGFβ receptor 1	Forward: GGCCGGGCCACAAAC
		Reverse: AAA​CAC​TGT​AAT​GCC​TTC​GCC
*Tgfbr2*	TGFβ receptor 2	Forward: ACG​TTC​CCA​AGT​CGG​ATG​TG
		Reverse: TTC​AGT​GGA​TGG​ATG​GTC​CT
*Tnfrsf1a*	TNF receptor 1	Forward: AAA​GGG​CAC​CTT​TAC​GGC​TT
		Reverse: ACC​TGG​GAC​ATT​TCT​TTC​CGA
*Tnfrsf1b*	TNF receptor 2	Forward: TAA​GTG​TCC​TCC​TGG​CCA​AT
		Reverse: CCT​GGG​TAT​ACA​TGC​TTG​CCT
*Vim*	Vimentin	Forward: CGG​AAA​GTG​GAA​TCC​TTG​CAG​G
		Reverse: AGC​AGT​GAG​GTC​AGG​CTT​GGA​A

### RNA-Seq and Bioinformatic Analysis

Total RNA was submitted to RNA-seq analysis by the WELGENE company, Taiwan. Fragments per kilobase per million (FPKM) reads of control (shCtrl) and glucocorticoid receptor knockdown (shGR1 and shGR2) were used to identify differentially expressed genes in the glucocorticoid receptor knockdown vs. control cells. To exclude the sequencing background, genes with FPKM less than 0.3 (Ramsköld et al., 2009) were defined as “non-expressed” genes and were excluded from the bioinformatic analysis. Genes with FPKM greater than 0.3 under any experimental conditions were selected for further analysis. Positive regulators for the vasopressin responses are genes that were downregulated in the glucocorticoid receptor knockdown vs. control cells, that is, genes with FPKM ratios (shGR/shCtrl) less than 1. Negative regulators for the vasopressin responses are genes that were upregulated in the glucocorticoid receptor knockdown vs. control cells, that is, genes with FPKM ratios (shGR/shCtrl) greater than 1. Kyoto Encyclopedia of Genes and Genomes (KEGG) pathway analysis was conducted using the DAVID Bioinformatics Resources (DAVID) (Huang et al., 2009a; Huang et al., 2009b). Chi-squared tests were performed to evaluate the significance of the regulated pathways.

### Immunoblotting

The cell proteins were dissolved in a protein sample buffer (1% Triton X-100, 50 mM Tris, 150 mM NaCl, 2 mM EDTA, and 0.5% SDC) with protease and phosphatase inhibitors. The protein concentrations were measured with bicinchoninic acid (Cat. 23225, Thermo-Fisher, United States). In general, 20 μg protein was mixed with 5X loading buffer (7.5% SDS, 30% glycerol, 50 mM Tris, pH 6.8, 200 mM DTT (dithiothreitol) and bromophenol blue a few), separated on a 10% SDS-PAGE gel at 15 mA, 160 V in 1X SDS-PAGE running buffer (25 mM Tris, 192 mM glycine, and 0.1% SDS) for 100 min. The separated proteins in the gel were transferred to a nitrocellulose membrane (Cat. 10600004, GE Healthcare Life Science, United States) in 1X Fairbank buffer (25 mM Tris, 192 mM glycine, and 20% (V/V) methanol) with 200 mA for 1 h. The membrane was incubated on a shaker at room temperature for 1 h with blocking buffer 0.1% BSA in 1X TBS-T (20 mM Tris, 150 mM NaCl, and 0.1% Tween 20). After the removal of the blocking buffer, the membrane was incubated overnight with the primary antibody diluted in the blocking buffer. The antibodies were as follows: α-SMA (Cat. 14968, α-smooth muscle actin) and phospho-ERK1/2 (Cat. 4370) from Cell Signaling, United States; α-tubulin (Cat. GTX112141) and GR (Cat. GTX101120) from GeneTex, Taiwan; Akt1/2/3 (Cat. sc-8312), AQP2 (Cat. sc-9880), ERK (Cat. sc-153), phospho-Akt (Cat. sc-7958-R), and vimentin (Cat. sc-373717) from Santa Cruz, United States; or β-actin (Cat. A5441) from Sigma-Aldrich, United States. The next day, the membrane was washed three times (10 min each) with TBS-T on a shaker and incubated with the secondary antibody (diluted in the blocking buffer) for 1 h on a shaker at room temperature. The secondary antibodies were as follows: IRDye 800 goat anti-rabbit (Cat. 926–32211), IRDye 800 donkey anti-goat (Cat. 926–32214), or IRDye 680 goat anti-mouse (Cat. 926–68020) from LI-COR, United States. Finally, the membrane was washed three times with TBS-T before visualization and quantification using a near-infrared fluorescence Odyssey scanner and software (LI-COR, United States). The choice for loading control, β-actin, or α-tubulin depended on the molecular weights of the target proteins and the species of the antibodies.

### Surface Biotinylation

After experiments, the cells on Transwell^®^ were first incubated at 4°C for 20 min to minimize cellular activities including vesicular trafficking. The apical sides of the cells were washed with cold PBS-CM (1 mM MgCl_2_ and 0.1 mM CaCl_2_ in 1X PBS (phosphate-buffered saline), pH 6.4) three times prior to incubation for 30 min at 4°C with 20 mM NaIO_4_ (in PBS-CM) to oxidize glycosylated proteins on the cell surface. After three washes with cold PBS-CM, the cells were incubated with 5 mM biocytin hydrazide in the apical side (Cat. 28020, Thermo-Fisher, United States) to label oxidized apical surface proteins with biotin. After the labeling, 50 mM NH_4_Cl was added to the apical side to stop the reaction. Thereafter, both apical and basolateral sides were washed with PBS-CM three times and continued to immunofluorescence confocal microscopy.

### Immunofluorescence Confocal Microscopy

The mpkCCD cells grown on Transwell^®^ were washed with ice-cold PBS-CM three times prior to fixation with 4% paraformaldehyde (in PBS-CM) for 20 min at room temperature. The cells were then washed with PBS-CM three times before being treated with membrane permeabilization buffer (0.3% Triton X-100, 0.1% BSA (bovine serum albumin), and 1 mM NaN_3_ in 1X PBS) for 30 min at room temperature. To block non-specific binding, the cells were incubated with IF blocking buffer (1% BSA, 0.05% saponin, 0.2% gelatin, and 1 mM NaN_3_ in 1X PBS) for 30 min at room temperature before being incubated with the primary AQP2 antibody (Wang et al., 2017) at 4°C overnight. The surface-labeled biotin was stained using Alexa Fluor™ 594 streptavidin (Cat. S32356, Invitrogen, United States). After washing with IF washing buffer (0.1% BSA, 0.05% saponin, 0.2% gelatin, and 1 mM NaN_3_ in 1X PBS) three times, the cells were incubated with the secondary antibody for 1 h at room temperature. The cell nuclei were stained with DAPI (4′,6-diamidino-2-phenylindole, 1 μg/ml in 1X PBS) at room temperature for 10 min. After two washes with PBS-CM, the cells were mounted in a fluorescence mounting medium (Cat. S3023, Agilent Technologies, United States) and covered with a cover glass. Confocal images were acquired using a Zeiss LSM880 microscope and processed by ZEN Blue software. Quantification of the images was performed by Zen software (Black edition, Carl Zeiss Microscopy, United States). For colocalization measurements of two proteins, the fluorescence signals from each protein were determined with a set threshold value based on background noise, that is, no primary antibody (or no phalloidin) staining control. Colocalization was calculated as a percentage of the pixels that are doubly positive for two proteins divided by the pixels that were positive for one protein.

## Results

### RNA-Seq Transcriptomic Analysis Revealed Pathways Regulated by the Glucocorticoid Receptor

**FIGURE 1:**
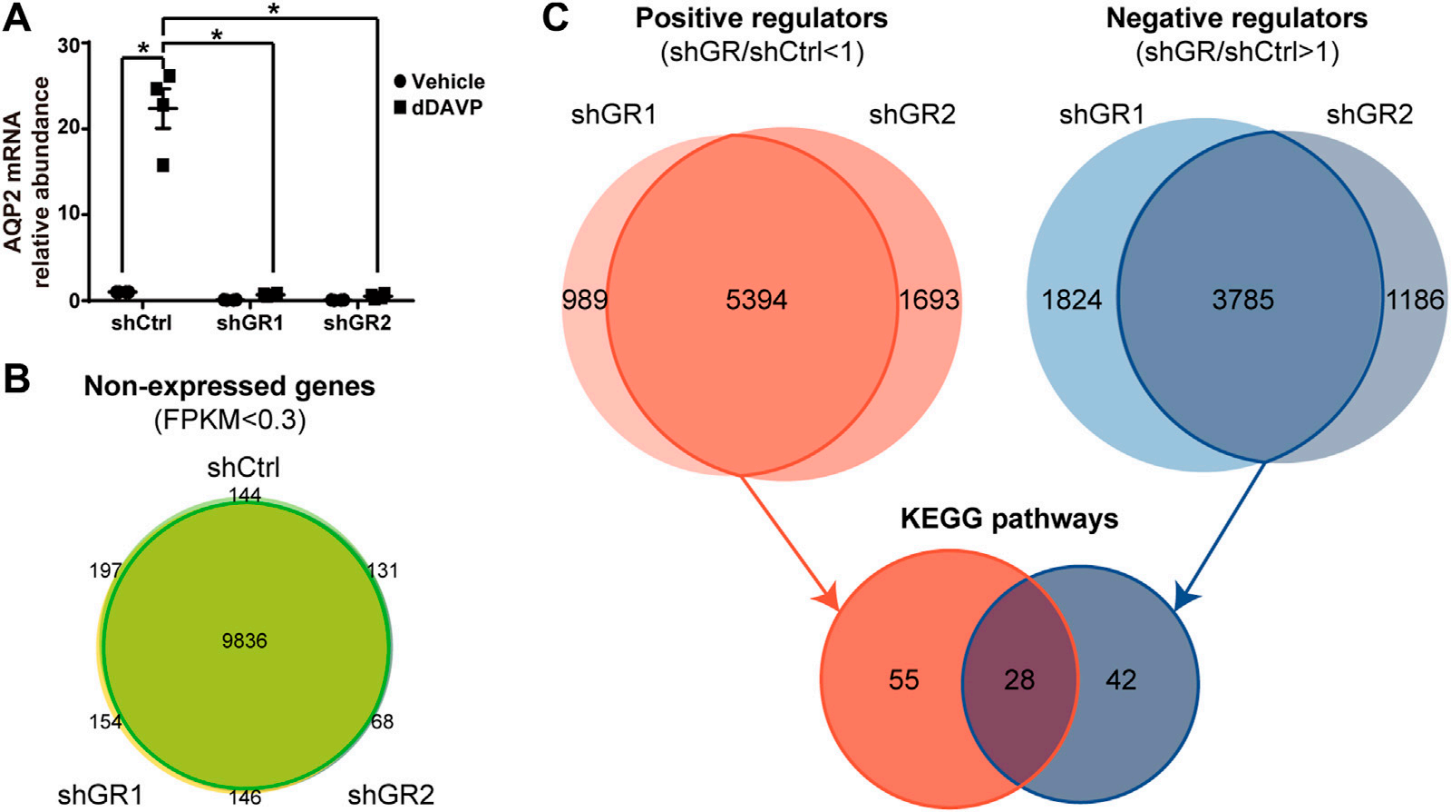
Glucocorticoid receptor knockdown altered the transcriptomic landscape of the mpkCCD cells under the vehicle conditions. **(A)** Quantitative RT-PCR measurements of the AQP2 mRNA level in control (shCtrl) and glucocorticoid receptor knockdown (shGR1 and shGR2) cells in response to vehicle vs. 1 nM vasopressin analog dDAVP for 24 h. **(B)** Venn diagram summary of the non-expressed genes in the shCtrl, shGR1, and shGR2 cells based on RNA-seq. Non-expressed genes are those with FPKM (Fragments Per Kilobase of transcript per Million) value <0.3 under all experimental conditions. **(C)** Venn diagram summary of positive and negative regulators for the *Aqp2* gene expression. Positive regulators are downregulated genes with lower FPKM in the shGR vs. shCtrl cells (shGR < shCtrl). Negative regulators are upregulated genes with higher FPKM in the shGR vs. shCtrl cells (shGR > shCtrl). Positive regulators (red line enclosed) and negative regulators (blue line enclosed) common to shGR1 and shGR2 cells were subjected to KEGG pathway analysis using the DAVID Bioinformatics.

To identify pathways regulated by the glucocorticoid receptor for the vasopressin-induced *Aqp2* gene expression (Kuo et al., 2018), we compared transcriptomes of the mpkCCD cells with or without stable glucocorticoid receptor knockdown (Ho et al., 2021). Figure 1A characterizes the responses of these cells to the vasopressin V2 receptor-specific analog dDAVP vs. vehicle. In the control cells without glucocorticoid receptor knockdown, dDAVP induced a 22.5-fold increase in AQP2 mRNA compared to unity under the vehicle conditions (Figure 1A, shCtrl, vehicle vs. dDAVP). The dDAVP-induced increases in AQP2 mRNA were diminished in the glucocorticoid receptor knockdown cells with two different small-hairpin sequences (Figure 1A, shGR1 and shGR2, vehicle vs. dDAVP), confirming the effects of glucocorticoid receptor knockdown on the vasopressin-induced *Aqp2* gene expression (Ho et al., 2021). RNA-seq was performed to analyze the transcriptomes of these cells (shCtrl, shGR1, and shGR2) under the vehicle conditions (i.e., without dDAVP stimulation). A total of 22,025 genes were quantified. Among them, 9,836 genes with an FPKM <0.3 under all experimental conditions (i.e., shCtrl, shGR1, and shGR2 cells under vehicle condition) were considered non-expressed (Figure 1B) (Ramsköld et al., 2009). Furthermore, 5,394 and 3,785 genes showed decreases and increases, respectively, in the transcript abundance in the glucocorticoid receptor knockdown cells (Figure 1C). The 5,394 decreased genes were considered the positive regulators of vasopressin responses because their decreases reduced the vasopressin-induced *Aqp2* gene expression. Table 2 shows 55 KEGG pathways associated with these positive regulators including “vasopressin-regulated water reabsorption”. The 3,785 increased genes were considered the negative regulators of vasopressin responses as their increases reduced the vasopressin-induced *Aqp2* gene expression. Table 3 shows 42 KEGG pathways associated with these negative regulators including the “TNF signaling pathway” and “TGFβ signaling pathway.” Chi-squared tests assured statistical significance in the alterations of the aforementioned three KEGG pathways in the glucocorticoid receptor knockdown cells (Table 4).

**TABLE 2:** KEGG pathway analysis of the positive regulators.

	Count	%	*p*-value
2-Oxocarboxylic acid metabolism	10	0.2	3.10E-02
Adipocytokine signaling pathway	26	0.5	4.80E-02
Alzheimer’s disease	82	1.6	2.10E-09
Amyotrophic lateral sclerosis (ALS)	20	0.4	4.00E-02
Arginine biosynthesis	10	0.2	3.10E-02
Bacterial invasion of epithelial cells	33	0.6	1.70E-03
Biosynthesis of amino acids	39	0.7	3.00E-06
Biosynthesis of antibiotics	94	1.8	3.80E-09
Carbon metabolism	63	1.2	6.90E-11
Central carbon metabolism in cancer	26	0.5	1.10E-02
Circadian rhythm	16	0.3	4.60E-03
Citrate cycle (TCA cycle)	17	0.3	2.30E-03
Collecting duct acid secretion	14	0.3	8.70E-03
Fatty acid elongation	17	0.3	1.00E-04
Fructose and mannose metabolism	17	0.3	4.90E-03
Glutathione metabolism	23	0.4	1.20E-02
Glycerophospholipid metabolism	35	0.7	1.20E-02
Glycine, serine, and threonine metabolism	21	0.4	6.70E-04
Glycolysis/gluconeogenesis	26	0.5	1.60E-02
Glycosaminoglycan biosynthesis - chondroitin sulfate/dermatan sulfate	10	0.2	4.40E-02
Glycosylphosphatidylinositol (GPI)-anchor biosynthesis	16	0.3	2.50E-04
Glyoxylate and dicarboxylate metabolism	15	0.3	6.30E-03
HIF-1 signaling pathway	39	0.7	4.90E-03
Huntington’s disease	87	1.7	1.50E-08
Inositol phosphate metabolism	35	0.7	2.20E-05
Insulin resistance	40	0.8	1.10E-02
Lysosome	56	1.1	1.50E-06
Metabolic pathways	503	9.6	5.30E-35
Metabolism of xenobiotics by cytochrome P450	24	0.5	3.80E-02
N-Glycan biosynthesis	26	0.5	9.60E-05
Non-alcoholic fatty liver disease (NAFLD)	80	1.5	1.00E-11
Other glycan degradation	11	0.2	6.00E-03
Oxidative phosphorylation	77	1.5	9.90E-14
Parkinson’s disease	75	1.4	1.00E-10
Pentose phosphate pathway	16	0.3	3.10E-03
Peroxisome	32	0.6	1.00E-02
Phosphatidylinositol signaling system	42	0.8	1.80E-04
Proximal tubule bicarbonate reclamation	11	0.2	3.20E-02
Purine metabolism	62	1.2	5.20E-03
Pyruvate metabolism	18	0.3	9.80E-03
Regulation of autophagy	18	0.3	1.90E-05
Ribosome	94	1.8	2.60E-23
SNARE interactions in vesicular transport	17	0.3	3.40E-03
Sphingolipid metabolism	24	0.5	5.90E-04
Sphingolipid signaling pathway	45	0.9	7.30E-03
Sulfur metabolism	7	0.1	1.10E-02
Sulfur relay system	9	0.2	4.60E-04
Synaptic vesicle cycle	26	0.5	6.70E-03
Thyroid hormone signaling pathway	39	0.7	3.40E-02
Toxoplasmosis	36	0.7	4.00E-02
Tuberculosis	57	1.1	3.00E-02
Valine, leucine, and isoleucine degradation	27	0.5	3.40E-04
Vasopressin-regulated water reabsorption	20	0.4	5.40E-03
VEGF signaling pathway	24	0.5	1.80E-02

**TABLE 3:** KEGG pathway analysis of the negative regulators.

	Count	%	*p*-value
Adherens junction	24	0.6	1.70E-03
Amino sugar and nucleotide sugar metabolism	15	0.4	3.40E-02
Axon guidance	31	0.8	5.00E-02
B-cell receptor signaling pathway	21	0.6	1.30E-02
Bladder cancer	13	0.4	4.00E-02
Cell cycle	59	1.6	1.90E-14
Chagas disease (American trypanosomiasis)	27	0.7	2.60E-02
Colorectal cancer	21	0.6	4.40E-03
Cytosolic DNA-sensing pathway	17	0.5	7.60E-02
DNA replication	15	0.4	1.30E-03
Dorso-ventral axis formation	11	0.3	6.20E-03
Epstein–Barr virus infection	40	1.1	6.30E-04
Fanconi anemia pathway	27	0.7	3.60E-08
Focal adhesion	51	1.4	7.00E-03
GnRH signaling pathway	22	0.6	7.20E-02
Hepatitis B	45	1.2	8.40E-05
Hepatitis C	35	0.9	1.40E-02
Herpes simplex infection	51	1.4	7.70E-03
Hippo signaling pathway	39	1.1	8.80E-03
Homologous recombination	15	0.4	7.20E-05
HTLV-I infection	68	1.8	1.70E-03
Leishmaniasis	18	0.5	4.10E-02
Lysine degradation	19	0.5	1.90E-03
MAPK signaling pathway	65	1.8	5.70E-04
Mismatch repair	10	0.3	7.80E-03
mRNA surveillance pathway	40	1.1	5.20E-08
Oocyte meiosis	38	1	3.50E-05
Osteoclast differentiation	32	0.9	2.30E-02
PI3K-Akt signaling pathway	80	2.2	6.30E-03
Progesterone-mediated oocyte maturation	23	0.6	3.80E-02
Ras signaling pathway	54	1.5	1.30E-02
Renal cell carcinoma	24	0.6	7.10E-04
Ribosome biogenesis in eukaryotes	30	0.8	7.80E-05
RNA degradation	32	0.9	7.30E-06
RNA transport	58	1.6	1.70E-07
Spliceosome	54	1.5	4.90E-10
Steroid biosynthesis	9	0.2	9.80E-03
T-cell receptor signaling pathway	26	0.7	3.60E-02
Terpenoid backbone biosynthesis	9	0.2	3.40E-02
TGF-beta signaling pathway	27	0.7	1.80E-03
TNF signaling pathway	32	0.9	2.50E-03
Viral carcinogenesis	54	1.5	1.60E-02

**TABLE 4:** Chi-squared tests of selected KEGG pathways regulated by glucocorticoid receptor.

1. Vasopressin-regulated water reabsorption pathway		
	Downregulated gene numbers	Not Downregulated gene numbers
Genes that belong to the pathway	11	11
Genes that do not belong to the pathway	3,774	20,911
The Chi-squared statistic is 20.415 with a *p*-value <0.00001.		
**2. TNF signaling pathway**		
	**Upregulated gene numbers**	**Not upregulated gene numbers**
Genes that belong to the pathway	30	62
Genes that do not belong to the pathway	5,364	19,251
The Chi-squared statistic is 6.2845 with a *p*-value of 0.01218.		
**3. TGFβ signaling pathway**		
	**Upregulated gene numbers**	**Not upregulated gene numbers**
Genes that belong to the pathway	18	35
Genes that do not belong to the pathway	5,374	19,278

The Chi-square statistic is 4.5854 with a p-value of 0.032245.

### Quantitative RT-PCR Confirmed Partly the RNA-Seq Results

**FIGURE 2:**
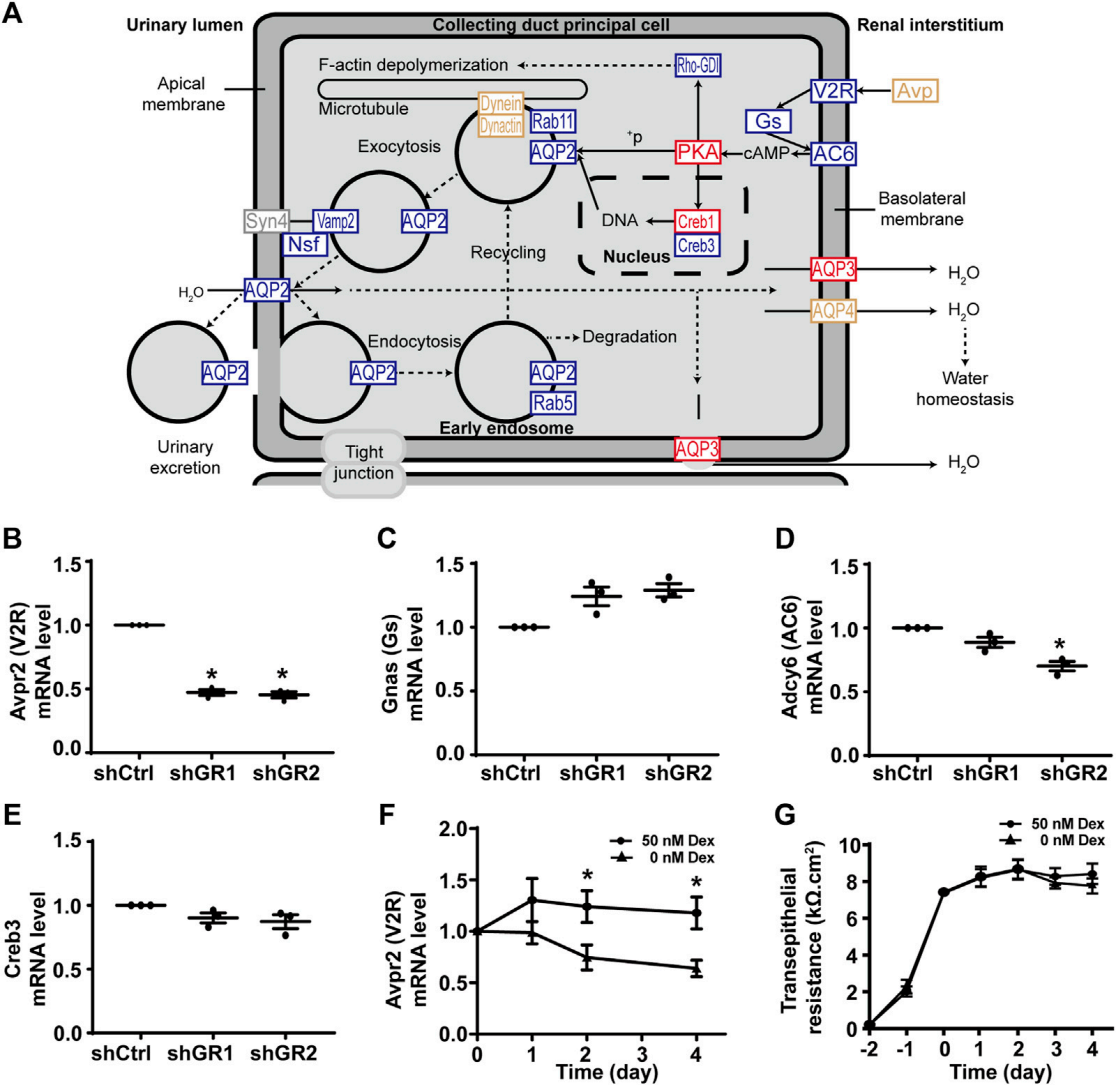
Glucocorticoid receptor knockdown suppressed the KEGG “vasopressin-regulated water reabsorption” pathway in the mpkCCD cells. **(A)** KEGG “vasopressin-regulated water reabsorption” pathway. Genes are abbreviated and color-coded: blue, downregulated; gray: not found in the RNA-seq data; red, upregulated; yellow, no change in the glucocorticoid receptor knockdown cells. ^+^P, phosphorylate. **(B–E)** Quantitative RT-PCR measurements of Avpr2, Gnas, Adcy6, and Creb3 mRNA in control (shCtrl) and glucocorticoid receptor knockdown (shGR1 and shGR2) cells under vehicle conditions. Values are mean ± S. E. summarized from three independent experiments. Asterisks indicate statistical significance (*p* < 0.05, *t*-test). **(F)** Avpr2 mRNA level and **(G)** transepithelial electrical resistance of polarized mpkCCD cells exposed to 0 vs. 50 nM dexamethasone (Dex).

Figure 2A plots the “vasopressin-regulated water reabsorption” KEGG pathway. Among the 22 components in the pathway, 11 of them showed decreases in transcript abundance in the glucocorticoid receptor knockdown cells by RNA-seq analysis. Table 5 lists all components in the pathway. Also, four of them were elected for quantitative RT-PCR validation: vasopressin V2 receptor (Avpr2), guanine nucleotide-binding protein G(s) subunit α (Gnas), adenylyl cyclase type 6 (Adcy6), and cyclic AMP-responsive element-binding protein 3 (Creb3). Quantitative RT-PCR showed reduction in the Avpr2 transcript level in two glucocorticoid receptor knockdown cells (Figure 2B, shGR1 and shGR2), and reduction in the Adcy6 transcript level in one glucocorticoid receptor knockdown cell (Figure 2D, shGR2). No change was found in the Gnas (Figure 2C) and Creb3 (Figure 2E) transcript levels in the glucocorticoid receptor knockdown cells. The previous observations suggest that the glucocorticoid receptor maintains the Avpr2 expression. This was reassured by the expression of Avpr2 in the cells maintained in the presence of the glucocorticoid receptor agonist dexamethasone (Figure 2F, 50 vs. 0 nM Dex). Dexamethasone removal gradually reduced the Avpr2 mRNA levels without affecting cell polarization as the transepithelial resistance was similar regardless of dexamethasone (Figure 2G).

**TABLE 5:** “Vasopressin-regulated water reabsorption pathway” gene table.

Symbol on graph	Official gene name	NCBI gene ID	log_2_ (shGR/shCtrl)
Upregulated genes
AQP3	*Aqp3*	11828	0.477
PKA	*Prkaca*	18747	0.136
Creb1	*Creb1*	12912	0.11
Dynein	*Dync1i2*	13427	0.09
Dynein	*Dync1li2*	234663	0.3
Dynein	*Dync2h1*	110350	0.125
Downregulated genes
V2R	*Avpr2*	12000	-1.198
AC6	*Adcy6*	11512	-0.467
Rho-GDI	*Arhgdia*	192662	-0.166
AQP2	*Aqp2*	11827	-2.91
Rab11	*Rab11a*	53869	-0.174
Rab5	*Rab5a*	271457	-0.172
Vamp2	*Vamp2*	22318	-0.333
Nsf	*Nsf*	18195	-0.128
Dynein	*Dync2li1*	213575	-0.303
Dynein	*Dync1li1*	235661	-0.077
Creb3	*Creb3*	12913	-0.105
Unchanged genes
Avp	*Avp*	11998	0
AQP4	*Aqp4*	11829	N/A
Dynactin	*Dctn1*	13191	N/A
Dynein	*Dync1h1*	13424	N/A
Dynein	*Dync1i1*	13426	FPKM<0.3

**FIGURE 3:**
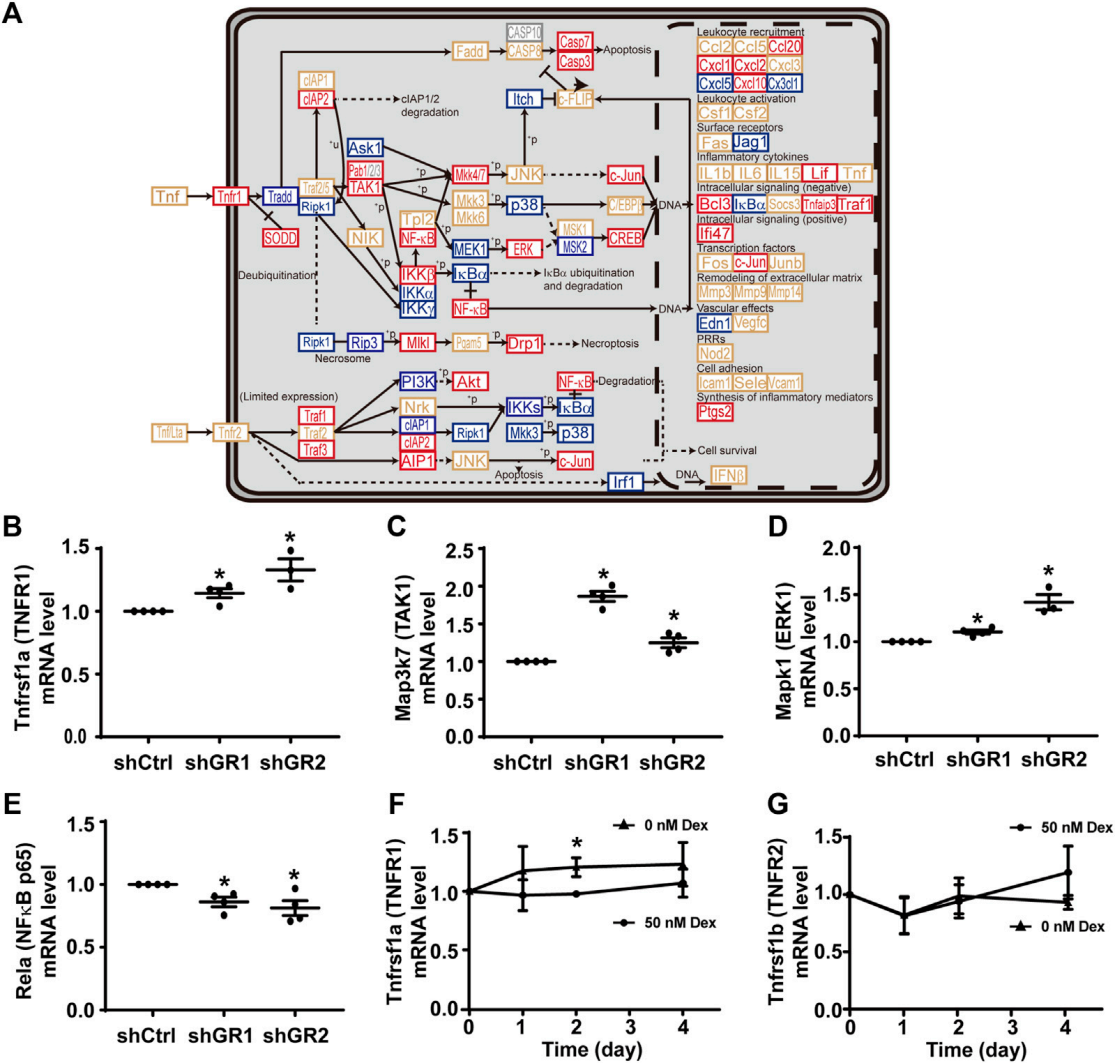
Glucocorticoid receptor knockdown enhanced the KEGG “TNF signaling pathway” in the mpkCCD cells. **(A)** KEGG “TNF signaling pathway.” Genes are abbreviated and color-coded: blue, downregulated; gray: not found in the RNA-seq data; red, upregulated; yellow, no change in the glucocorticoid receptor knockdown cells. ^+^P, phosphorylate; ^−^P, dephosphorylate; PRRs, pattern recognition receptors; ^+^u, ubiquitinylate. **(B–E)** Quantitative RT-PCR measurements of Tnfrsf1a, Map3k7, Mapk1, and Rela mRNA levels in control (shCtrl) and glucocorticoid receptor knockdown (shGR1 and shGR2) cells under vehicle conditions. Values are mean ± S. E. summarized from three or four independent experiments. Asterisks indicate statistical significance (*p* < 0.05, *t*-test). **(F,G)** Quantitative RT-PCR measurements of Tnfrsf1a and Tnfrsf1b mRNA levels in polarized mpkCCD cells exposed to 0 vs. 50 nM dexamethasone (Dex).

Figure 3A plots the “TNF signaling pathway.” Among the 92 components in the pathway, 30 of them showed increases in the transcript abundance in the glucocorticoid receptor knockdown cells by RNA-seq analysis. Table 6 lists all components in the TNF signaling pathway. Also, four of them were elected for quantitative RT-PCR validation: tumor necrosis factor receptor superfamily member 1A (Tnfrsf1a also Tnfr1), mitogen-activated protein kinase 7 (Map3k7 also known as TAK1), mitogen-activated protein kinase 1 (Mapk1 also known as ERK1), and transcription factor p65 (Rela also known as NFκB p65). Consistent with the RNA-seq data, quantitative RT-PCR measurements showed increases in the Tnfrsf1a (Figure 3B), Map3k7 (Figure 3C), and Mapk1 (Figure 3D) mRNA levels in the glucocorticoid receptor knockdown cells. Quantitative RT-PCR measurement of Rela mRNA levels in the glucocorticoid receptor knockdown cells did not agree with the RNA-seq data (Figure 3E). Thus, the RNA-seq and quantitative RT-PCR data are grossly consistent with the suppression of the TNF signaling pathway by the glucocorticoid receptor, although removal of dexamethasone did not elevate the Tnfrsf1a (Figure 3F) or Tnfrsf1b (Figure 3G) mRNA level.

**TABLE 6:** “TNF signaling pathway” gene table.

Symbol on graph	Official gene name	NCBI gene ID	log_2_ (shGR/shCtrl)
Upregulated genes		
Tnfr1	*Tnfrsf1a*	21937	0.085
SODD	*Bag4*	67384	0.625
cIAP2	*Birc3*	11796	0.592
Traf1	*Traf1*	22029	2.61
Traf3	*Traf3*	22031	0.498
Pab1	*Pabpc1*	18458	0.016
TAK1	*Map3k7*	26409	0.115
IKKβ	*Ikbkb*	16150	0.05
Mlkl	*Mlkl*	74568	0.344
AIP1	*Wdr1*	22388	0.149
Mkk4	*Map2k4*	26398	0.161
Akt1	*Akt1*	11651	0.116
ERK	*Mapk1*	26413	0.025
Drp1	*Dnm1l*	74006	0.126
Casp3	*Casp3*	12367	0.518
Casp7	*Casp7*	12369	0.447
c-Jun	*Jun*	16476	0.978
CREB	*Creb1*	12912	0.109
Ccl20	*Ccl20*	20297	4.173
Cxcl1	*Cxcl1*	14825	1.346
Cxcl2	*Cxcl2*	20310	1.07
Cxcl10	*Cxcl10*	15945	1.612
Lif	*Lif*	16878	0.728
Bcl3	*Bcl3*	12051	0.255
Tnfaip3	*Tnfaip3*	21929	0.423
Traf1	*Traf1*	22029	2.61
Ifi47	*Ifi47*	15953	0.974
Ptgs2	*Ptgs2*	19225	0.39
NFkB	*Rela*	19697	0.167
Mkk7	*Map2k7*	26400	0.013
Downregulated genes
Tradd	*Tradd*	71609	−0.154
Ripk1	*Ripk1*	19766	−0.112
Ask1	*map3k5*	26408	−0.503
Rip3	*Nrip3*	78593	−1.428
IKKα	*chuk*	12675	−0.076
IKKγ	*Ikbkg*	16151	−0.337
Mkk3	*Map2k3*	26397	−0.46
MEK1	*Map2k1*	26395	−0.209
IkBa	*Nfkbia*	18035	−0.923
Ripk1	*Ripk1*	19766	−0.112
Itch	*Itch*	16396	−0.555
p38	*Mapk14*	26416	−0.219
MSK2	*Rps6ka4*	56613	−0.258
Irf1	*Irf1*	16362	−0.3544
Cxcl5	*Cxcl5*	20311	−1.234
Cx3cl1	*Cx3cl1*	20312	−0.137
Jag1	*Jag1*	16449	−0.866
Edn1	*Edn1*	13614	−0.299
NFκB	*Nfkb1*	18033	−0.25
NFκB	*Rel*	19696	−0.03
NFκB	*Nfkb2*	18034	−0.22
PI3K	*Pik3r1*	18708	−0.293
Unchanged genes
Nod2	*Nod2*	257632	FPKM<0.3
Icam1	*Icam1*	15894	FPKM<0.3
Sele	*Sele*	20339	0
Vcam1	*Vcam1*	22329	FPKM<0.3
IFNb	*Ifnb1*	15977	0
NFkB	*Relb*	19698	N/A
Mkk6	*Map2k6*	26399	FPKM<0.3
Lta	*Lta*	16992	FPKM<0.3
Tnfr2	*Tnfrsf1b*	21938	FPKM<0.3
MSK1	*Rps6ka5*	73086	N/A
cIAP1	*Birc2*	11797	N/A

**FIGURE 4:**
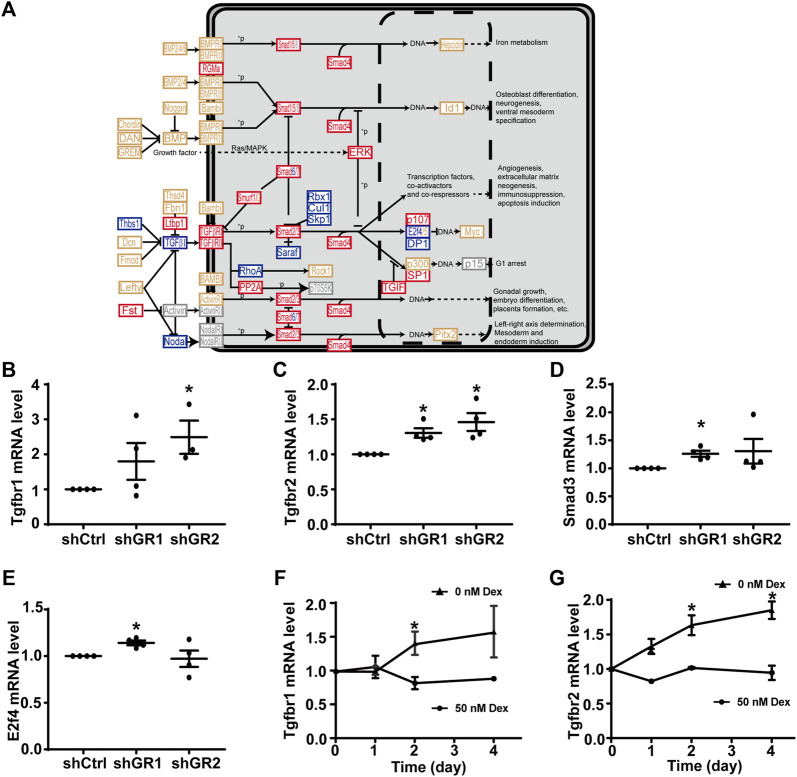
Glucocorticoid receptor knockdown enhanced the KEGG “TGFβ signaling pathway” in the mpkCCD cells. **(A)** KEGG “TGFβ signaling pathway.” Genes are abbreviated and colored: blue, downregulated; gray: not in RNA-seq data; red, upregulated; yellow, no change in the glucocorticoid receptor knockdown cells. ^+^P, phosphorylate; ^−^P, dephosphorylate. **(B–E)** Quantitative RT-PCR measurements of Tgfbr1, Tgfbr2, Smad3, and E2f4 mRNA levels in control (shCtrl) and glucocorticoid receptor knockdown (shGR1 and shGR2) cells under vehicle conditions. Values are mean ± S. E. summarized from three or four independent experiments. Asterisks indicate statistical significance (*p* < 0.05, *t*-test). **(F,G)** Quantitative RT-PCR measurements of Tgfbr1 and Tgfbr2 mRNA levels in polarized mpkCCD cells exposed to 0 vs. 50 nM dexamethasone (Dex).

Figure 4A plots the “TGFβ signaling pathway.” Among the 53 components in the pathway, 18 of them showed increases in the transcript abundance in the glucocorticoid receptor knockdown cells by RNA-seq analysis. Table 7 lists all components in the TGFβ signaling pathway. Also, four of them were elected for quantitative RT-PCR validation: TGFβ receptor type-1 (Tgfbr1), TGFβ receptor type-2 (Tgfbr2), mothers against decapentaplegic homolog 3 (Smad3), and transcription factor E2F4 (E2f4). Consistent with the RNA-seq data, quantitative RT-PCR measurements showed increases in the Tgfbr1 (Figure 4B) and Tgfbr2 (Figure 4C) mRNA levels in one or two glucocorticoid receptor knockdown cells and increases in the Smad3 (Figure 4D, shGR1) mRNA level in one glucocorticoid receptor knockdown cell. Quantitative RT-PCR measurement of E2f4 mRNA level in one glucocorticoid receptor knockdown cell did not agree with the RNA-seq data (Figure 4E, shGR1). Thus, the quantitative RT-PCR data are mostly consistent with the RNA-seq data and suggest the suppression of the TGFβ signaling pathway by the glucocorticoid receptor. In line with this, the removal of dexamethasone elevated both Tgfbr1 (Figure 4F) and Tgfbr2 (Figure 4G) mRNA levels.

**TABLE 7:** “TGFβ signaling pathway” gene table.

Symbol on graph	Official gene name	NCBI gene ID	log_2_ (shGR/shCtrl)
Upregulated genes			
Fst	*Fst*	14313	0.486
Ltbp1	*Ltbp1*	268977	2.071
RGMa	*Rgma*	244058	0.717
TGFβRI	*Tgfbr1*	21812	0.308
TGFβRII	*Tgfbr2*	21813	0.489
Smurf1	*Smurf1*	75788	0.247
PP2A	*Ppp2ca*	19052	0.052
Smad1	*Smad1*	17125	0.332
Smad2	*Smad2*	17126	0.185
Smad3	*Smad3*	17127	0.331
Smad4	*Smad4*	17128	0.052
Smad5	*Smad5*	17129	0.075
Smad7	*Smad7*	17131	0.683
ERK	*Mapk1*	26413	0.025
p107	*Rbl1*	19650	0.194
SP1	*Sp1*	20683	0.15
BMP	*Bmp1*	12153	0.406
TGIF	*Tgif1*	21815	0.322
Downregulated genes			
TGFβ1	*Tgfb1*	21803	-0.075
RhoA	*Rhoa*	11848	0.036
Saraf	*Saraf*	67887	-0.256
Rbx1	*Rbx1*	56438	-0.1
Cul1	*Cul1*	26965	-0.067
Skp1	*Skp1*	21402	-0.544
E2f4	*E2f4*	104394	-0.218
DP1	*Tfdp1*	21781	-0.125
Thbs1	*Thbs1*	21825	-0.462
Smad6	*Smad6*	17130	-0.713
Unchanged genes			
Lefty	*Lefty1*	13590	0
Thsd4	*Thsd4*	207596	FPKM<0.3
Bmp2	*Bmp2*	12156	FPKM<0.3
Bmp4	*Bmp4*	12159	FPKM<0.3
Bmp6	*Bmp6*	12161	FPKM<0.3
Noggin	*Nog*	18121	FPKM<0.3
Fbn1	*Fbn1*	14118	FPKM<0.3
Nodal	*Nodal*	18119	0
BMPRI	*Bmpr1a*	12166	N/A
BMPRII	*Bmpr1b*	12167	FPKM<0.3
ActivinR1	*Acvr1*	11477	N/A
Rock1	*Rock1*	19877	N/A
Hepcidin	*Hamp*	84506	0
Id1	*Id1*	15901	N/A
p300	*Ep300*	328572	N/A
Pitx2	*Pitx2*	18741	FPKM<0.3
Myc	*Myc*	17869	N/A
Chordin	*Chrd*	12667	FPKM<0.3
DAN	*Nbl1*	17965	N/A
GREM	*Grem1*	23892	FPKM<0.3
Dcn	*Dcn*	13179	N/A
Fmod	*Fmod*	14264	FPKM<0.3
Smurf2	*Smurf2*	66313	N/A
Bambi	*Bambi*	68010	FPKM<0.3
E2f5	*E2f5*	13559	FPKM<0.3

### TNF and TGFβ Reduced the Vasopressin-Induced *Aqp2* Gene Expression

**FIGURE 5:**
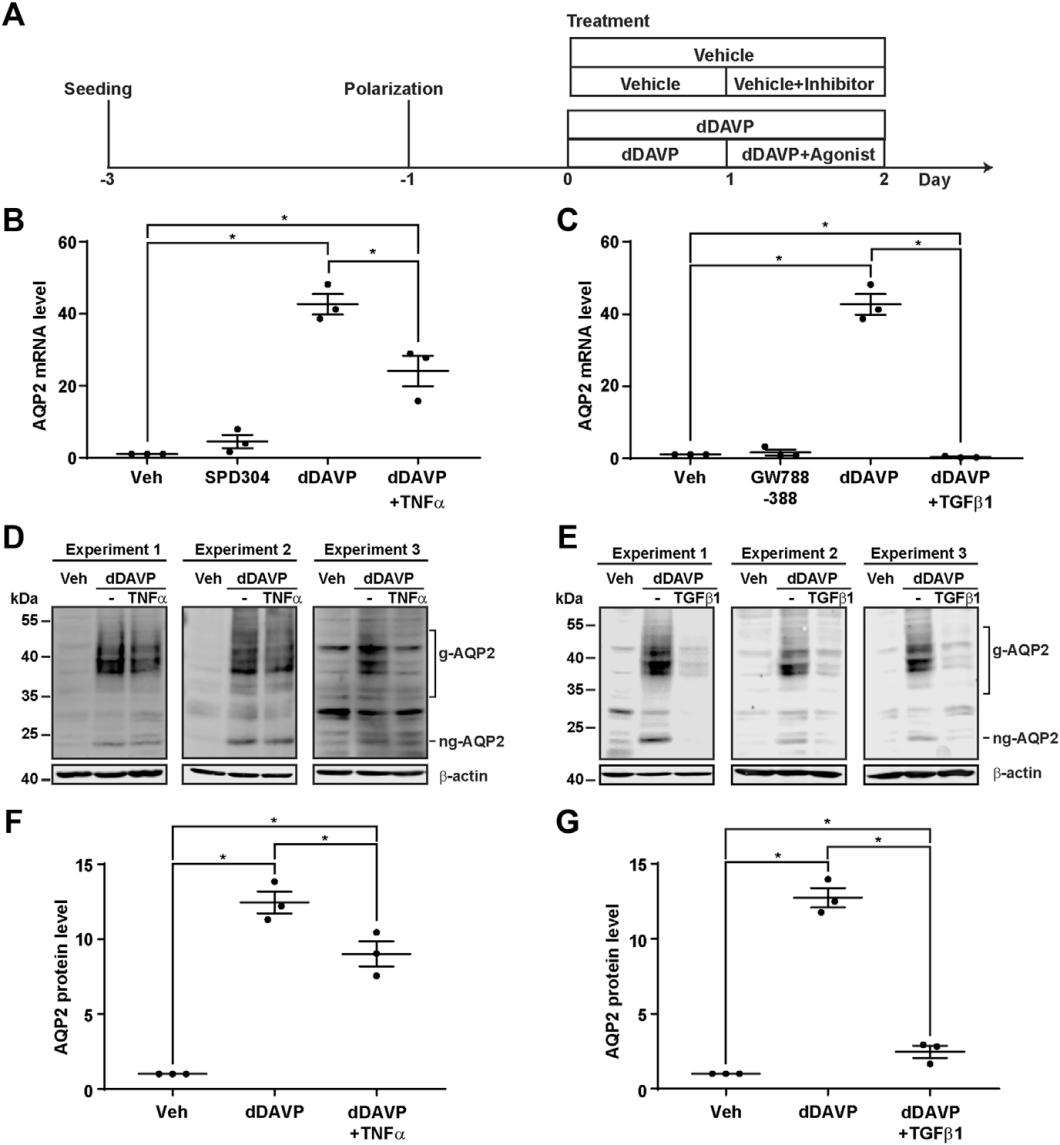
TNFα and TGFβ1 reduced the vasopressin-induced *Aqp2* gene expression. **(A)** Experimental protocol. **(B,C)** Quantitative RT-PCR measurements of the AQP2 mRNA level in the mpkCCD cells exposed 24 h to the TNF receptor inhibitor (100 nM SPD304), TGFβ receptor inhibitor (10 μM GW788388), dDAVP (1 nM), dDAVP + TNFα (40 ng/ml), or dDAVP + TGFβ1 (10 ng/ml). **(D,E)** Immunoblots and **(F,G)** summaries of immunoblotting results for the AQP2 protein level in the mpkCCD cells under the same conditions as for AQP2 mRNA measurements. Values are mean ± S. E. summarized from three independent experiments. Values were normalized with loading control (β-actin) before being compared against those under the vehicle conditions. Asterisks indicate statistical significance (*p* < 0.05, *t*-test). g-AQP2, glycosylated AQP2; ng-AQP2, non-glycosylated AQP2; Veh, vehicle.

The negative regulatory role of the TNF signaling pathway and the TGFβ signaling pathway in the vasopressin responses prompted us to examine whether inhibition of the TNF or TGFβ pathway would induce the *Aqp2* gene expression in the absence of vasopressin (Figure 5A). Quantitative RT-PCR measurements of the AQP2 mRNA level showed that the TNF activity inhibitor (SPD304) or TGFβ receptor inhibitor (GW788388) alone did not induce the AQP2 mRNA expression in the absence of dDAVP (Figures 5B,C). In contrast, TNF significantly reduced (Figure 5B) whereas TGFβ1 profoundly suppressed (Figure 5C) dDAVP-induced increases in the AQP2 mRNA levels. The reduction in the AQP2 mRNA level manifested the reduction in the AQP2 protein level (Figures 5D–G). Thus, both TNF and TGFβ signaling pathways suppressed the dDAVP-induced *Aqp2* gene expression. Of the two, the TGFβ signaling pathway plays a key negative regulatory role in the vasopressin-induced *Aqp2* gene expression.

### TGFβ Did Not Induce Epithelial-To-Mesenchymal Transition

**FIGURE 6:**
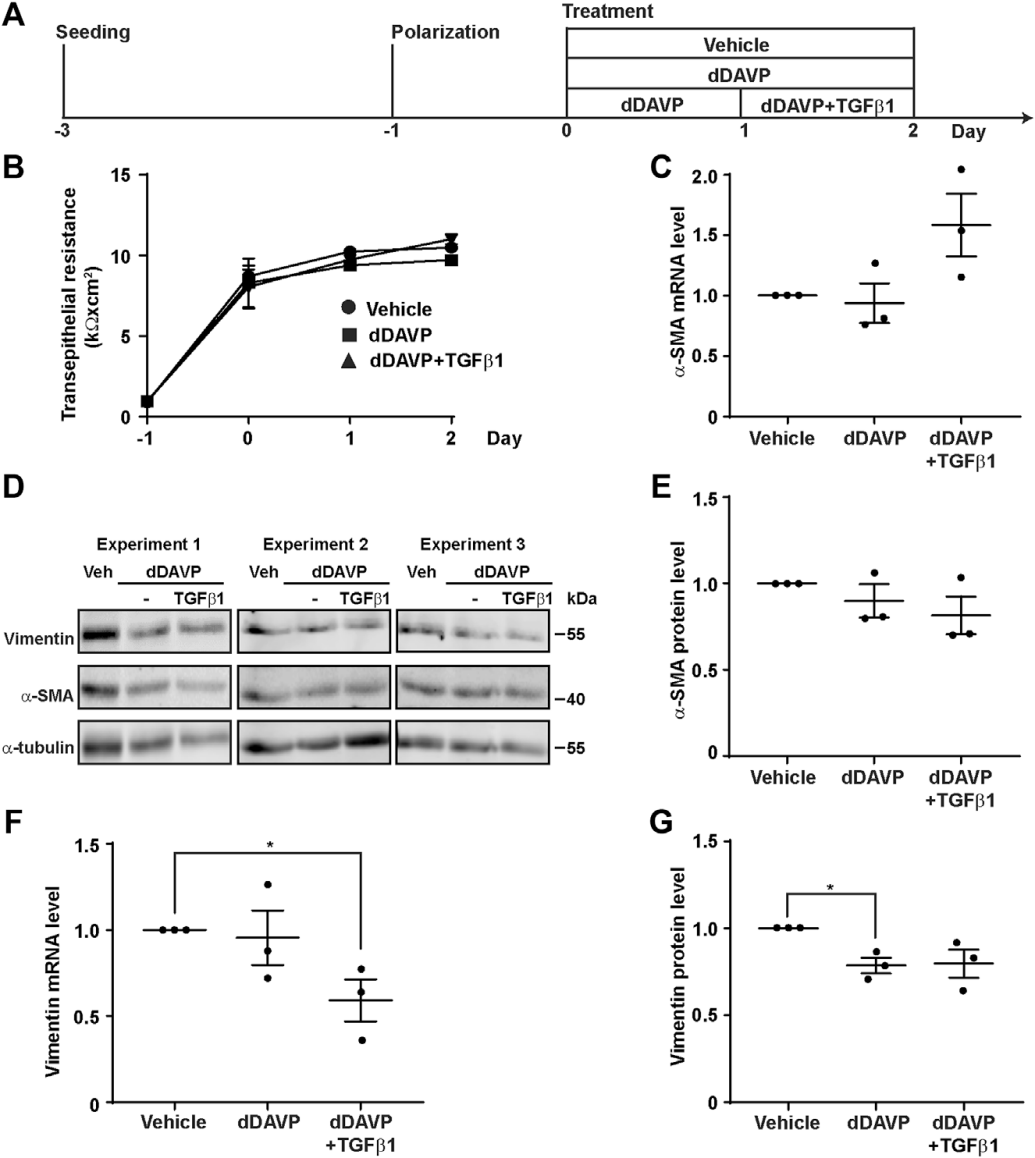
TGFβ1 did not induce epithelial-to-mesenchymal transition. **(A)** Experimental protocol. **(B)** Transepithelial electrical resistance of mpkCCD cells grown on Transwell^®^ membrane under the vehicle, dDAVP (1 nM), or dDAVP + TGFβ1 (10 ng/ml) conditions. **(C,F)** Quantitative RT-PCR measurements of α-SMA and vimentin mRNA levels in the mpkCCD cells under the same conditions. **(D)** Immunoblots and **(E,G)** summaries of immunoblotting results for α-SMA and vimentin protein levels in the cells under the same conditions. Values are mean ± S. E. summarized from three independent experiments. Values were normalized with loading control (α-tubulin) before being compared against those under the vehicle conditions. Asterisks indicate statistical significance (*p* < 0.05, *t*-test).

TGFβ is known to induce epithelial-to-mesenchymal transition, a condition that would induce dedifferentiation of the collecting duct cells and render them irresponsive to vasopressin (Schnaper et al., 2009). To test whether TGFβ induces epithelial-to-mesenchymal transition to suppress the vasopressin-induced *Aqp2* gene expression in the mpkCCD cells, the markers of the epithelial-to-mesenchymal transition, namely, α-SMA and vimentin, as well as transepithelial resistance, were measured (Figure 6A). The transepithelial resistances were similar under all experimental conditions (Figure 6B, vehicle, dDAVP, or dDAVP plus TGFβ1), indicating that TGFβ1 did not alter epithelial integrity. Likewise, the α-SMA mRNA and protein levels were not altered under all experimental conditions (Figures 6C–E). The effects on the vimentin mRNA and protein levels were inconsistent with the epithelial-to-mesenchymal transition. During epithelial-to-mesenchymal transition, vimentin was expected to increase (Vuoriluoto et al., 2011), which was not observed. Compared to the vehicle control, dDAVP did not alter the vimentin mRNA level (Figure 6F). TGFβ1 with dDAVP reduced it (Figure 6F). Compared to the vehicle control, dDAVP slightly reduced the vimentin protein level (Figures 6D,G). TGFβ1 with dDAVP did not alter it (Figures 6D,G). Overall, the previous observations were not consistent with the epithelial-to-mesenchymal transition in the mpkCCD cells induced by TGFβ1 in the presence of dDAVP.

### TGFβ Did Not Affect Vasopressin-Induced Apical AQP2 Trafficking

**FIGURE 7:**
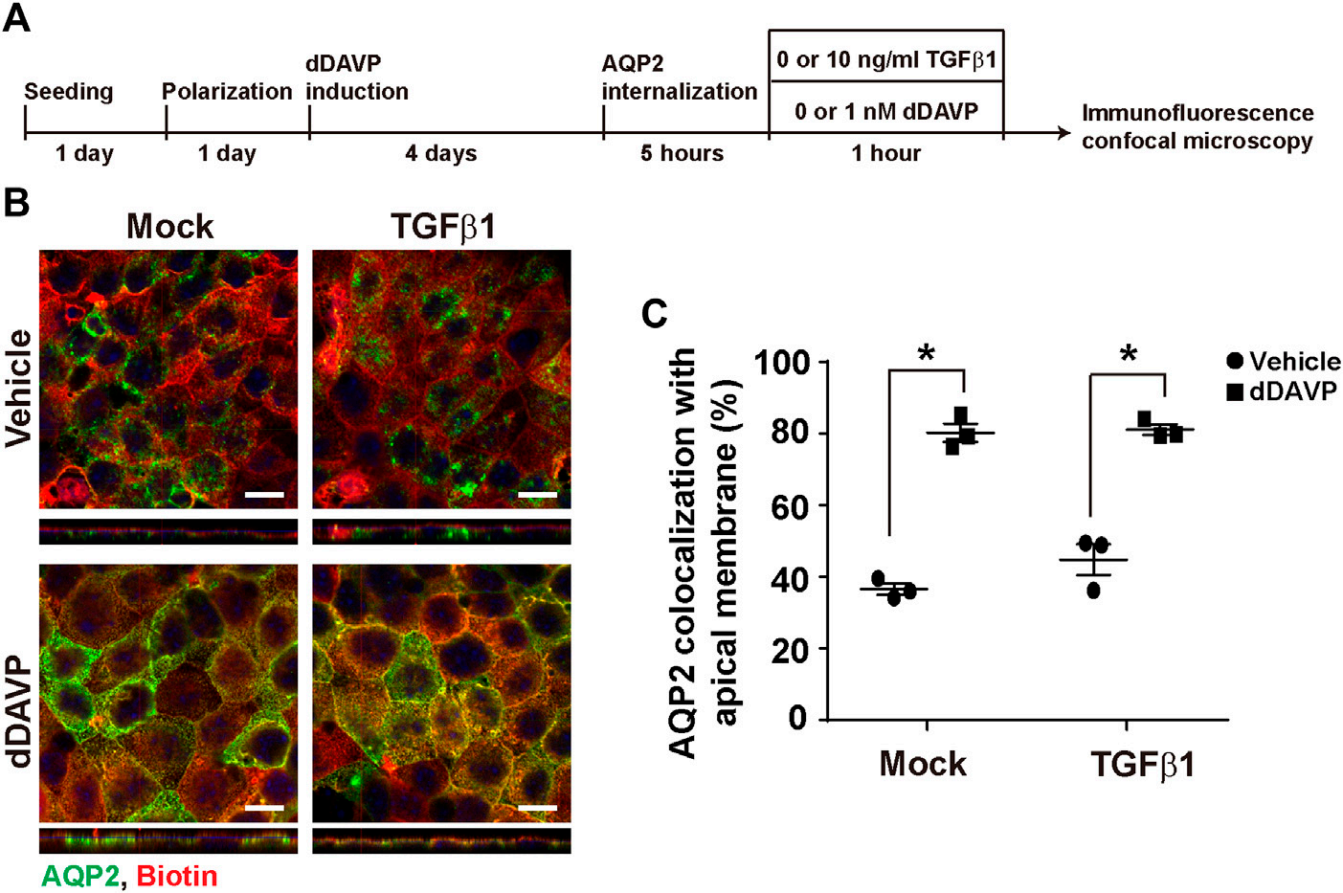
TGFβ1 did not affect vasopressin-induced apical AQP2 trafficking. **(A)** Experimental protocol. **(B)** Representative confocal immunofluorescence micrographs of AQP2 (green) and surface-biotinylated apical membrane (red) of mpkCCD cells in response to vehicle vs. dDAVP (1 nM, 1 h) in the absence or presence of TGFβ1 (10 ng/ml). Scale bars represent 10 μm. **(C)** Summary of the imaging results. Values are mean ± S. E. summarized from three independent experiments. Asterisks indicate statistical significance (*p* < 0.05, *t*-test).

To test whether TGFβ affects vasopressin-induced apical AQP2 trafficking, polarized mpkCCD cells were exposed to vehicle vs. dDAVP in the absence or presence of TGFβ1 prior to confocal immunofluorescence microscopy (Figure 7A). As seen in Figure 7B, AQP2 was intracellular in the absence of TGFβ1 under the vehicle conditions. Upon dDAVP stimulation, AQP2 was detected at the apical membrane that was delineated with surface biotin (Figure 7B). On average, about 36.5% AQP2 was apical in the mpkCCD cells in the absence of TGFβ1 under the vehicle conditions (Figure 7C). Upon dDAVP stimulation, about 80.3% AQP2 was in the apical plasma membrane, indicating vasopressin-induced apical AQP2 translocation. Similar observations were made in the presence of TGFβ1. Thus, TGFβ1 did not affect vasopressin-induced apical AQP2 trafficking in the mpkCCD cells.

### TGFβ Reduced Vasopressin-Induced Akt Activation

**FIGURE 8:**
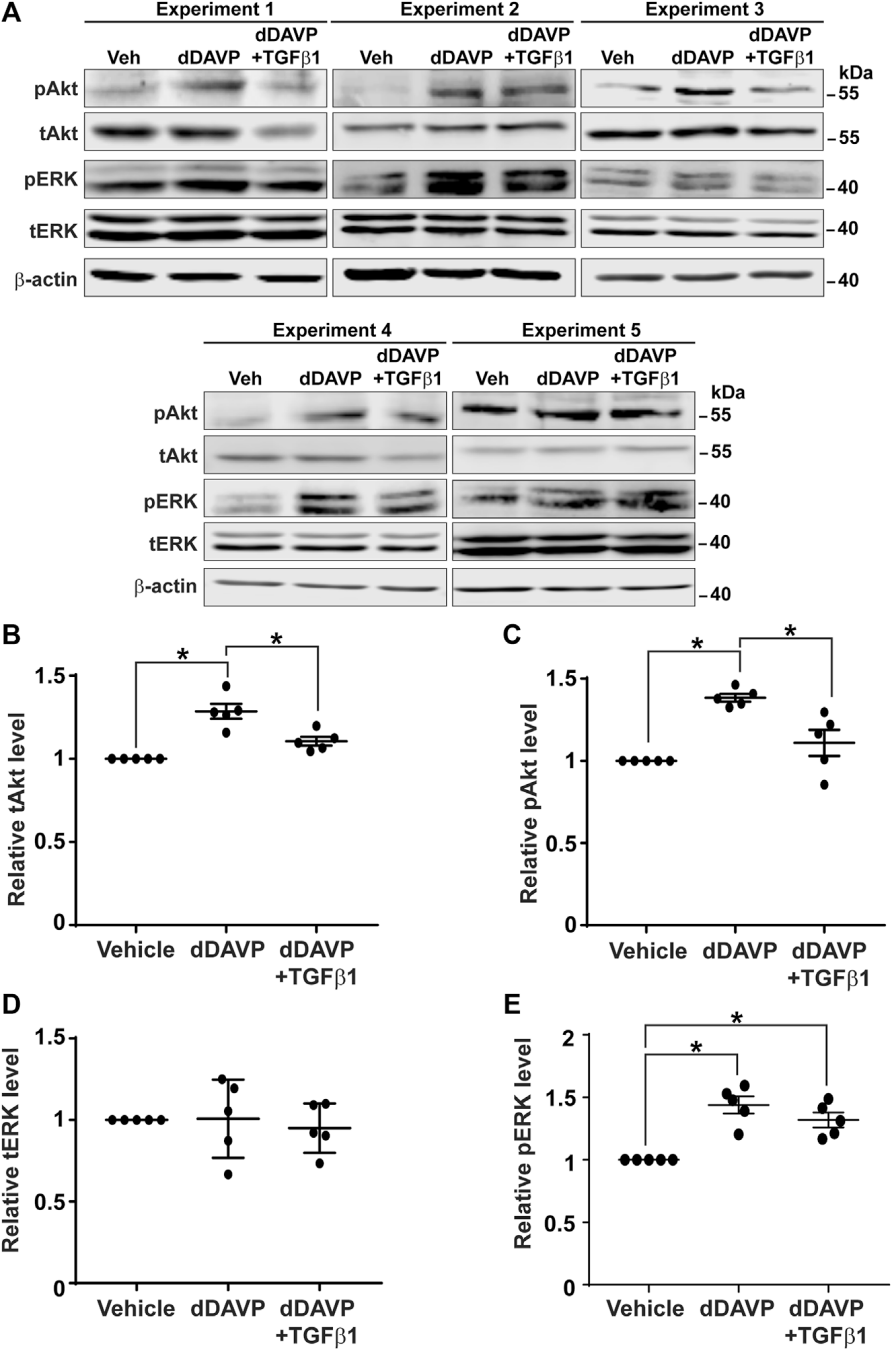
TGFβ1 reduced vasopressin-induced Akt activation. **(A)** Immunoblots for total and phosphorylated Akt and Erk in the mpkCCD cells in response to vehicle vs. dDAVP (1 nM, 24 h) in the absence or presence of TGFβ1 (10 ng/ml). **(B–E)** Summary of the immunoblotting results. Values are mean ± S. E. summarized from five independent experiments. Values were normalized with loading control (β-actin) before being compared against those under the vehicle conditions. Asterisks indicate statistical significance (*p* < 0.05, *t*-test). pAkt, phosphorylated Akt; pErk, phosphorylated Erk; tAkt, total Akt; tErk, total Erk.

Akt and Erk are two protein kinases common to both the vasopressin signaling pathway and TGFβ pathway (Vander Ark et al., 2018). It was of interest to test whether TGFβ1 reduces vasopressin-induced increases in Akt and Erk activation (Pisitkun et al., 2008), thereby diminishing the vasopressin-induced *Aqp2* gene expression. Immunoblotting showed that dDAVP increased phosphorylation and the total protein abundance of Akt and that TGFβ1 reduced these increases (Figure 8A). On average, dDAVP increased Akt phosphorylation and abundance by 38.5 and 28.6%, respectively (Figures 8B,C). Both were significantly reduced by TGFβ1. DDAVP did not affect Erk abundance (Figures 8A,D) but increased its phosphorylation level (Figures 8A,E). TGFβ1 did not reduce dDAVP-induced Erk phosphorylation (Figures 8A,E). TGFβ1 did not alter Erk total protein abundance (Figures 8A,D). Thus, TGFβ1 reduced vasopressin-induced Akt activation.

## Discussion

System tools have been instrumental in identifying novel regulatory pathways for complex physiological processes (Rinschen et al., 2018). Common practices to examine the functions of the identified pathways include chemical alterations of the entire pathway or gene-specific manipulation of key nodes in the pathways. Gene-specific manipulation *via* knockout provides an unequivocal functional evaluation of the identified pathways (Limbutara et al., 2019; Datta et al., 2020; Isobe et al., 2020; Raghuram et al., 2020). In cases when gene-specific knockout results in cell or embryonic lethality, gene-specific knockdown provides another means for evaluation (Wang et al., 2017; Lin et al., 2019; Wang et al., 2020; Wong et al., 2020). Oftentimes, gene-specific manipulation involves several generations of selection that could result in alteration in the transcriptomic landscape and complicate the evaluation. The present study represents such an example. Previous system approaches have alluded to the role of glucocorticoid receptor in the *Aqp2* gene expression in the kidney collecting duct cells (Yu et al., 2009; Kikuchi et al., 2021). In line with this, glucocorticoid receptor agonist dexamethasone increases the vasopressin-induced *Aqp2* gene expression in the collecting duct mpkCCD cells (Kuo et al., 2018). The stable glucocorticoid receptor knockdown blunted the vasopressin-induced *Aqp2* gene expression in the cells (Ho et al., 2021). In the current study, we found that the transcriptome was significantly altered in the stable glucocorticoid receptor knockdown cells that do not respond to vasopressin (Figure 1). Most notably, the “vasopressin-regulated water reabsorption pathway” with positive regulatory roles in the vasopressin responses was suppressed in the glucocorticoid receptor knockdown cells (Figure 2). In addition, the “TNF signaling pathway” (Figure 3) and the “TGFβ signaling pathway” (Figure 4) with negative regulatory functions in the vasopressin responses were elevated in the glucocorticoid receptor knockdown cells (Lin et al., 2016; Lee et al., 2018). With the downregulation of a positive regulatory pathway and upregulation of two negative regulatory pathways, it came without a surprise that the stable glucocorticoid receptor knockdown cells did not respond to vasopressin. Thus, gene-specific manipulation is a powerful means to investigate the functions of a pathway; however, the results should be interpreted with caution, especially when stable gene-specific manipulation alters the transcriptome landscape.

In line with the suppression of the “vasopressin-regulated water reabsorption pathway” in the stable glucocorticoid receptor knockdown cells was the reduction in the vasopressin V2 receptor Avpr2 transcript level (Table 5; Figure 2B). In fact, the Avpr2 transcript was maintained at a certain level in the presence of the glucocorticoid receptor agonist dexamethasone (Figure 2F). The removal of dexamethasone reduced its level. One potential explanation is the transcriptional regulation of Avpr2 by the glucocorticoid receptor. The three glucocorticoid receptor-binding sites in the Avpr2 5’ flanking region were predicted commonly by three programs, namely, TRANSFAC^®^ (Wingender et al., 2018), PROMO (Briones-Orta et al., 2017), and TRAP (Manke et al., 2008): 1) −1,934 to −1,929, 2) −424 to −419, and 3) −248 to −243. Additional experiments are needed to test the possibility.

One explanation for the reduced *Aqp2* gene expression in the glucocorticoid receptor knockdown cells has to do with the interplay between AQP2 phosphorylation and ubiquitylation (Tamma et al., 2011). Short-chain ubiquitylation is involved in AQP2 endocytosis and degradation (Kamsteeg et al., 2006). AQP2 phosphorylation at serine 269 occurs in parallel with AQP2 ubiquitylation at the apical plasma membrane in the presence of vasopressin (Moeller et al., 2014). Serine 269 phosphorylation overrides ubiquitylation, keeping AQP2 from endocytosis (Moeller et al., 2014; Wang et al., 2017). Serine 261 phosphorylation occurs after AQP2 ubiquitylation and endocytosis (Tamma et al., 2011). Serine 261 phosphorylation was thought to stabilize ubiquitylated AQP2 from degradation (Tamma et al., 2011). Thus, glucocorticoid receptor knockdown could affect AQP2 phosphorylation and hence AQP2 stability and abundance. Given the low AQP2 abundance in the glucocorticoid knockdown cells (Figure 1A) (Ho et al., 2021), our attempt to measure AQP2 phosphorylation was of minimal success. In the knockdown cells with residual AQP2, we were able to measure AQP2 phosphorylation at serine 261, 264, and 269 in response to dDAVP by immunofluorescence confocal microscopy. AQP2 was apically localized in the knockdown cells in response to dDAVP (not shown). It responded to dDAVP with a decrease in 261 phosphorylation and increases in 264 and 269 phosphorylation, consistent with prior observations (Xie et al., 2010). Thus, the glucocorticoid receptor knockdown did not seem to affect AQP2 phosphorylation and thus ubiquitylation and degradation. However, the aforementioned statement was bound to one caveat, that is, knockdown efficiency. The cells with residual AQP2 might still have the glucocorticoid receptor that maintains proper vasopressin responses. In the cells where knockdown was complete, we could not measure AQP2 or its phosphorylation. Because we could not reach definitive conclusions, we reserved the aforementioned data.

Despite the drawbacks, stable gene-specific manipulation identified two pathways, namely, the “TNF signaling pathway” (Figure 3) and “TGFβ signaling pathway” (Figure 4) with pathophysiological relevance. Both pathways have negative regulatory roles in the vasopressin-induced *Aqp2* gene expression (Figure 5). TNF is a type II transmembrane protein produced primarily by immune cells (Dong et al., 2007). It functions as a homotrimer either in its membrane-bound form or it can be released as a soluble circulating polypeptide upon cleavage by a metalloproteinase called TNF-converting enzyme (Black et al., 1997). TNF is a central mediator of inflammation amongst a broad range of biological activities (Dong et al., 2007). A number of kidney injuries induce inflammation with elevated TNF levels, including acute kidney injury, renal ischemia/reperfusion injury, diabetic nephropathy, obstructive renal injury, and cisplatin-induced injury (Al-Lamki and Mayadas, 2015). In rats, cisplatin-induced kidney injury manifests reduced AQP2 abundance (Kim SW et al., 2001), in line with a negative regulatory role of TNF in the *Aqp2* gene expression. Conversely, the inhibition of TNF downstream effector interleukin 1β increases the *Aqp2* gene expression under ureteral obstruction conditions (Hu et al., 2019). Moreover, the transcript levels of TNF receptors (Tnfrsf1a and Tnfrsf21-23) are negatively correlated with the AQP2 transcript levels (Yu et al., 2009). All these observations are consistent with a pathophysiological mechanism by which TNF suppresses the vasopressin-induced *Aqp2* gene expression.

Mature TGFβ functions as a homodimer (Khalil, 1999). It binds to TGFβ receptor II that recruits TGFβ receptor I to regulate diverse biological processes (Massagué, 1996). TGFβ is a master regulator in renal inflammation and fibrosis, two major pathophysiological features of chronic kidney disease (Meng et al., 2016; Gu et al., 2020). Secreted by renal tubule cells and infiltrated macrophages, TGFβ causes tubular and glomerular epithelial-to-mesenchymal transition. This induces excessive production and deposition of the extracellular matrix in glomeruli and tubulointerstitium, leading to renal fibrosis and function loss (López-Hernández and López-Novoa, 2012; Meng et al., 2016; Tang et al., 2019; Gu et al., 2020). Patients in the early stages of chronic kidney disease are usually asymptomatic but may experience weakness related to anemia and polyuria (Romagnani et al., 2017), suggestive of the reduced *Aqp2* gene expression in the early stages. We found that TGFβ1 profoundly suppressed the vasopressin-induced *Aqp2* gene expression in the mpkCCD cells within 24 h (Figure 5). Thus, the TGFβ-suppressed *Aqp2* gene expression could account for polyuria experienced by patients in the early stages of chronic kidney disease. TGFβ receptor inhibitors and similarly TNF inhibitors could potentially alleviate the polyuric symptoms.

TGFβ-induced epithelial-to-mesenchymal transition does not seem to be the cause of the reduced *Aqp2* gene expression because TGFβ suppressed the vasopressin-induced *Aqp2* gene expression without inducing the epithelial-to-mesenchymal transition (Figures 5, 6). The epithelial-to-mesenchymal transition is a main issue that causes cultured collecting duct cells to lose their ability to respond to vasopressin and to express AQP2 (Yu et al., 2009; Li et al., 2015). When polarized on the Transwell^®^ membrane or homed to renal inner medullary collecting ducts, these cells reverse the epithelial-to-mesenchymal transition processes and regain the ability to respond to vasopressin with the *Aqp2* gene expression. The vasopressin escape and ureteral obstruction represent two pathophysiological states with the loss of the *Aqp2* gene expression associated with the induced epithelial-to-mesenchymal transition of the collecting duct principal cells (Stødkilde et al., 2011; Lee et al., 2018). In our experimental settings, TGFβ1 was able to reduce the vasopressin-induced *Aqp2* gene expression within 24 h without apparent epithelial-to-mesenchymal transition (Figures 5, 6). Thus, TGFβ likely exerts its effects *via* altering the vasopressin signaling network, that is, reducing vasopressin-induced Akt activation (Figure 8).

The effects of dexamethasone on the *Aqp2* gene expression in the collecting duct cells are complex, especially with systemic complications at the animal levels (Yasui et al., 1996; Saito et al., 2000; Kim JK et al., 2001; Chen et al., 2005). The evidence is strong that the glucocorticoid receptor agonist dexamethasone does not affect the *Aqp2* gene expression in rat cortical, outer, or inner medullary collecting ducts (Saito et al., 2000; Kwon et al., 2002; Li et al., 2008), albeit opposite observations under similar experimental settings (Chen et al., 2005). In fact, a single betamethasone injection was shown to increase the *Aqp2* gene expression in rats and increased their urine concentrating capacity (Yasui et al., 1996). Thus, there are complex systemic effects of dexamethasone on the *Aqp2* gene expression at the animal level. This is appreciated given that dexamethasone suppresses the vasopressin gene promoter activity in the hypothalamic cells (Kim JK et al., 2001), which, in turn, regulates the *Aqp2* gene expression in the kidney collecting duct cells (Moeller et al., 2013). Recently, we revisited the issues and found that dexamethasone increased the vasopressin-induced *Aqp2* gene expression in the mpkCCD cells (Kuo et al., 2018). Similar observations were made in the rat inner medullary collecting duct suspensions (Chen et al., 2015). Although we found that dexamethasone increased the vasopressin-induced *Aqp2* gene expression, the latter study found that dexamethasone reduced AQP2 protein degradation. Through different mechanisms, both studies are consistent with a positive regulatory role of glucocorticoid receptor in the amount of AQP2 protein. Indeed, glucocorticoid receptor knockdown blunted the vasopressin-induced *Aqp2* gene expression in the mpkCCD cells (Ho et al., 2021). Thus, mechanistic studies can benefit from representative collecting cell models, free from systemic complications in the animals.

In summary, gene-specific alteration is a powerful means to study biological pathways identified *via* system methods. Despite the risk of altering the transcriptomic landscape, gene-specific alteration can still identify regulatory pathways with pathophysiological significance, which might be exploited for medical benefits.

## Data Availability

The original contributions presented in the study are publicly available. This data can be found here: GEO accession: GSE194044, link: https://www.ncbi.nlm.nih.gov/geo/query/acc.cgi?acc=GSE194044. One may also access the summarized data set here: http://sbel.mc.ntu.edu.tw/mpkCCDTranscriptome_GR/mpkCCDTrGR.htm.
